# 

**Published:** 1883-05

**Authors:** 


CONTENTS.

ART. I.—Correlated Diseases of the Teeth and Ear. By Joseph Richardson, M. D., D. D. S.......1

ART. II.—Metallic Crowns. By William N. Morrison, D. D. S.......13

ART. III.—Meeting of State Boards of Dental Examiners. By Geo. Cushing D. D. S.......20

ART. IV.—Views about Decay of the Teeth—Old and New. By Dr. Mc Schlenker.......26

ART. V.—The Odontological Society of Great Britain.......31

ART. VI.—Odonto-Chirurgical Society of London.......36

ART. VII.—Keeping the Teeth Clean. By C. E. Francis, D. D. S.......38

ART. VIII.—Prof. Chisolm's Rules in Administering Chloroform.......41

EDITORIAL, ETC.

The Use of Narcotics.......43
Dr. Genese's Method of Treating Fractured Jaw.......44

MONTHLY SUMMARY.

Bone and Brain Disease in Syphilis.......46

A Remarkable Case.......47

Longevity of Occupations.......47

To Disguise the Odor of Iodiform.......48

Death from Dichloride of Ethidene.......48

Change of Time of Meeting of Southern Dental Association.......48

Meeting of Georgia State Dental Society.......48

